# *Tlx* Promotes Stroke-Induced Neurogenesis and Neuronal Repair in Young and Aged Mice

**DOI:** 10.3390/ijms252212440

**Published:** 2024-11-19

**Authors:** Dilaware Khan, Dagmar Bock, Hai-Kun Liu, Sajjad Muhammad

**Affiliations:** 1 Department of Neurosurgery, Medical Faculty, Heinrich-Heine-University, Moorenstrasse 5, 40225 Düsseldorf, Germany; 2 Next Generation Sequencing Core Facility, German Cancer Research Center (DKFZ), Im Neuenheimer Feld 280, 69120 Heidelberg, Germany; 3 Division of Molecular Neurogenetics, German Cancer Research Center (DKFZ), The DKFZ-ZMBH Alliance, Im Neuenheimer Feld 581, 69120 Heidelberg, Germany; l.haikun@dkfz.de; 4 Department of Neurosurgery, University of Helsinki and Helsinki University Hospital, 00014 Helsinki, Finland

**Keywords:** neural stem cells, *Tlx*, stroke, adult neurogenesis

## Abstract

Stroke is one of the leading causes of chronic disability in humans. It has been proposed that the endogenous neural stem/progenitor cells generate new neurons in the damaged area. Still, the contribution of these cells is negligible because a low number of newborn mature neurons are formed. *Tlx* conventional knock-out mice, *Tlx*-CreERT2 mice, and *Tlx*-overexpressing (*Tlx*-OE) mice were specifically chosen for their unique genetic characteristics, which were crucial for the experiments. Permanent and transient middle cerebral artery occlusion was used to induce stroke in the mice. Immunostainings for doublecortin and GFP/BrdU/NeuN were performed to study neurogenesis and fate mapping. The rotarod test was performed to assess motor deficits. Here, we show that stroke-induced neurogenesis is dramatically increased with the additional expression of two copies of the nuclear receptor-coding gene *tailless* (*Tlx*, also known as *Nr2e1*), which has been shown to be a master regulator of subventricular zone (SVZ) neural stem cells (NSCs). We show that *Tlx* expression is upregulated after stroke, and stroke-induced neurogenesis is blocked when *Tlx* is inactivated. *Tlx* overexpression in NSCs leads to massive induction of neurogenesis via stroke. More newborn mature neurons are formed in *Tlx*-overexpressing mice, leading to improved coordination and motor function recovery. Most importantly, we also demonstrate that this process is sustained in aged mice, where stroke-induced neurogenesis is nearly undetectable in wild-type animals. This study provides the first stem cell-specific genetic evidence that endogenous NSCs can be exploited by manipulating their master regulator, *Tlx*, and thus suggests a novel therapeutic strategy for neuronal repair.

## 1. Introduction

Stroke is the second leading cause of morbidity and mortality worldwide [1]. Ischemic stroke occurs when a vessel supplying blood supply to an area of the brain is blocked, causing brain damage and loss of neurological functions and consequently resulting in the most burdensome neurological disorders in terms of disability-adjusted life years [2]. Following a stroke, cognitive and motor function impairment demands therapeutic solutions to ameliorate the loss of these neurological functions. Neurogenesis offers a potential remedy to improve cognitive and motor functions after a stroke.

Neurogenesis persists in two regions in the adult mammalian brain: the SVZ of the lateral ventricle (LV) and the subgranular zone (SGZ) of the dentate gyrus (DG) [3,4]. The progeny of NSCs in the two regions follow their normal pathways in order to form new neurons and integrate them into the existing networks [5]. It has been shown that after brain injury (middle cerebral artery occlusion (MCAO)), neuroblasts are recruited from the neurogenic zones to the damaged area and form mature neurons in rats [6]. Many attempts have been made to increase neurogenesis via the infusion of factors, which may regulate NSC activity. However, those factors may also contribute to other processes. Thus, it is challenging to dissect their contribution to stroke-induced neurogenesis [7]. The contribution of endogenous NSCs to the repair of brain damage is controversial, in particular, because of the lack of well-established genetic models that specifically affect the NSC compartment. We have identified the nuclear receptor *Tlx* as a master regulator of adult SVZ neurogenesis. *Tlx* is exclusively expressed by type B cells (NSCs) in the adult mouse SVZ, and the inactivation of *Tlx* leads to the complete abolition of SVZ neurogenesis [8]. *Tlx* maintains adult neural stem cells in an undifferentiated proliferative state, and the cells lacking *Tlx* lose their ability to self-renew and proliferate [9]. Moreover, *Tlx* can attenuate oxidative stress [10] and dampen inflammation by inhibiting the NF-κB pathway and mitigating the pro-inflammatory markers [11]. Oxidative stress and neuroinflammation are known to suppress neurogenesis [12]. Additionally, *Tlx*, by improving SIRT1 [11], can be neuroprotective and contribute to neuronal plasticity [13]. Interestingly, we also found that the overexpression of *Tlx* in NSCs leads to a long-term increase in neurogenesis [14].

Here, we report that *Tlx* overexpression contributes to neurogenesis, increases cellular recovery, and improves motor function and coordination after experimental stroke in young and aged mice, suggesting *Tlx* to be a potential therapeutic target for cerebral ischemia.

## 2. Results

### 2.1. Tlx Is Required for Stroke-Induced Neurogenesis

**Figure 1:**
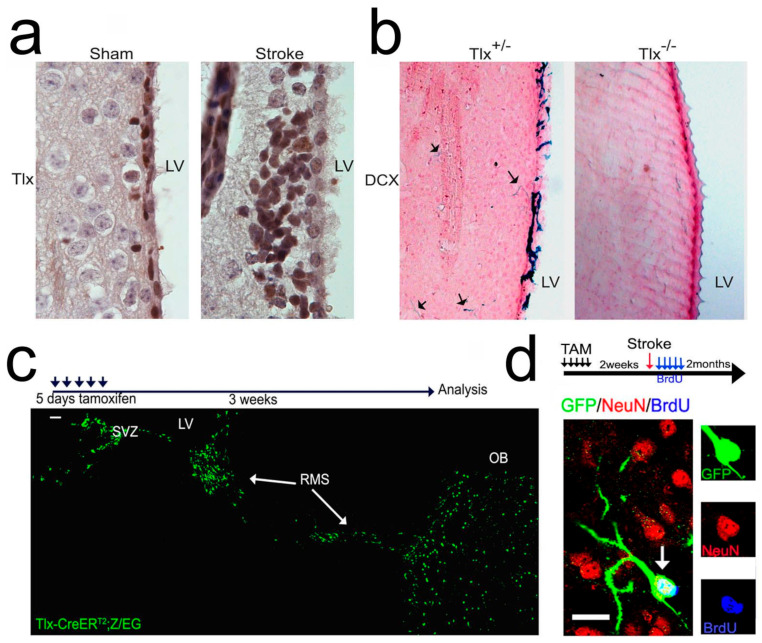
*Tlx* and *Tlx*-expressing cells are responsible for stroke-induced neurogenesis. (**a**) *Tlx* antibody staining of brain sections from sham (left) or stroke (right) WT mice 1 week after operation. (**b**) DCX antibody staining of brain sections from *Tlx^−/−^* and control (*Tlx*^+/−^) mice 1 week after stroke; arrows indicate individual DCX-positive cells in the striatum. Note that no DCX-positive cells are seen in the *Tlx^−/−^* brain sections. (**c**) GFP antibody staining of brain sections from *Tlx*-CreER^T2^;Z/EG mice treated as shown by the scheme. (**d**) GFP/NeuN/BrdU triple staining of brain sections from *Tlx*-CreER^T2^;Z/EG mice treated as shown by the scheme. Scale bar: 20 μm.

Here, we investigated the role of *Tlx* in stroke-induced neurogenesis by applying permanent cortical middle cerebral artery occlusion (pMCAO), which affects mainly the cortex and also induces SVZ neurogenesis [15,16]. We first asked whether the expression of *Tlx* is changed upon stroke, and therefore, wild-type (WT) mice were analyzed 1 week after a stroke. *Tlx* expression was strongly upregulated in the SVZ of mice with stroke compared to non-stroke sham-operated mice (Figure 1a), which suggests that *Tlx* was involved in the increase in SVZ neurogenesis after stroke. To investigate if *Tlx* was responsible for the process, we applied pMCAO to the *Tlx* knock-out (*Tlx*^−/−^) mice using *Tlx*-heterozygous mice (*Tlx*^+/−^) as the control. Since *Tlx*^−/−^ mice did not show any physiological neurogenesis in the adult SVZ [8,9], this experiment also gives direct evidence for the source of increased neurogenesis after stroke. We found doublecortin (DCX)-expressing cells, which migrated into the striatum of stroked *Tlx*^+/−^ animals (Figure 1b, arrows), which is in agreement with previously reported findings on SVZ neurogenesis after experimental stroke [17], but we did not observe DCX-expressing cells in *Tlx*^−/−^ mouse brains following stroke (Figure 1b). These findings suggest that the loss of *Tlx* expression resulted in a lack of adult SVZ neurogenesis.

To determine whether *Tlx*^+^ cells, the adult stem cells, significantly contribute to an increase in neurogenesis after stroke, we performed a genetic fate mapping experiment by crossing *Tlx*-CreER^T2^ mice with Z/EG reporter mice [18], which enabled us to genetically label the SVZ neurogenic system with GFP upon tamoxifen treatment [8]. The tamoxifen treatment of the *Tlx*-CreER^T2^;Z/EG mice led to a robust labeling of SVZ neural stem and progenitor cells with GFP (Figure 1c). We then performed stroke experiments on the tamoxifen-treated mice (2 weeks after treatment; see scheme in Figure 1d). GFP-positive cells were found in the striatum and cortex of the ipsilateral side but not in the contralateral side. GFP/BrdU/NeuN triple-positive cells were observed in the cortex 2 months after the stroke (Figure 1d). The cells developed with normal neuronal morphology. These fate mapping results directly demonstrate that *Tlx*-positive cells contributed to the increased neurogenesis and that their progeny could survive and differentiate into mature neurons in vivo.

### 2.2. Tlx Overexpression Led to an Increase in Stroke-Induced Neurogenesis from the SVZ

**Figure 2:**
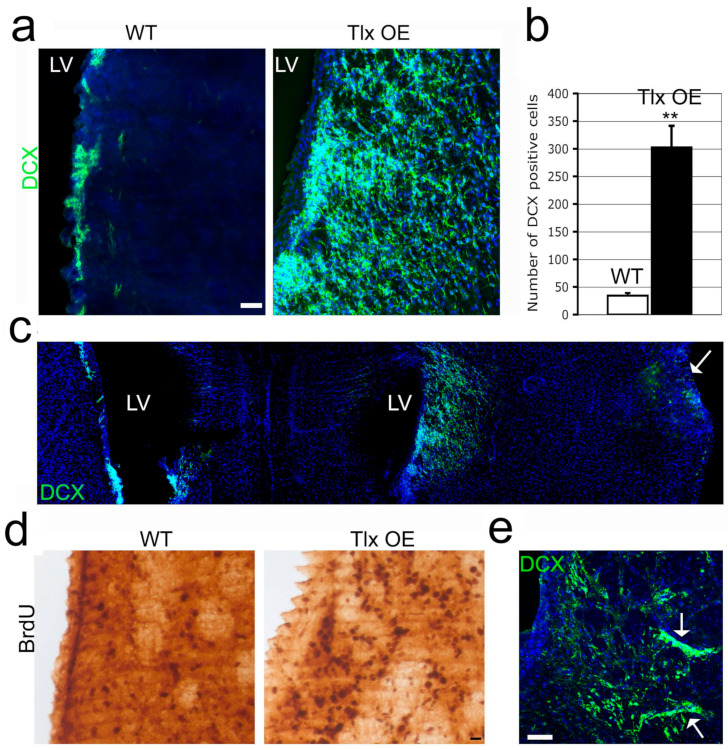
*Tlx* overexpression expands stroke-induced neurogenesis. (**a**) DCX antibody staining of brain sections from *Tlx*-OE mice 2 weeks after stroke. Note that only a few DCX-positive cells are found in WT brain sections. (**b**) Number of DCX-positive cells per striatum of brain sections from the stroke side (*n* = 6, mean ± standard deviation (SD), ** *p* < 0.01). (**c**) An overview of DCX staining in the *Tlx*-OE mouse brain sections. Note that stroke-induced neurogenesis is restricted to the stroke side (arrow) but not the collateral side. (**d**) BrdU IHC of brain sections from mice with stroke. BrdU was administrated daily for 5 days immediately after stroke. IHC was performed 2 days after the last BrdU injection. Note that more BrdU-positive cells are found in the striatum of *Tlx*-OE brain sections. (**e**) DCX staining of brain sections from *Tlx*-OE mice 2 weeks after stroke, arrows indicate clusters of DCX-positive cells. Scale bar: 20 μm.

We applied pMCAO to 2-month-old *Tlx*-OE mice. A dramatic increase in DCX-expressing cells was found on the stroke side of *Tlx*-OE mice 2 weeks after the stroke experiment (Figure 2a,b). We observed a massive increase in neuroblasts leaving the SVZ and migrating toward the lesion site. Importantly, we did not see any neuroblasts migrating to the contralateral side of stroked mice and non-stroked sham-operated mice (Figure 2c). We also performed BrdU administration as indicated in the scheme of Figure 1d. This allowed for the massive labeling of proliferating cells during 1 week following stroke. We found more BrdU-labeled cells in the SVZ and the adjacent striatum area of *Tlx*-OE mice, which indicates that more proliferation occurred in the neurogenic region of *Tlx*-OE mice (Figure 2d). Interestingly, the chain-like migratory structures formed by DCX-positive cells suggest that different migratory pathways were followed by these ectopic neuroblasts (Figure 2e, multiple arrows). To investigate whether the increase in stroke-induced neurogenesis in *Tlx*-OE mice also applies to other stroke models, we performed the half-hour transient MCAO, which limits the lesion to the striatum. More ectopic DCX^+^ cells were found in the striatum of *Tlx*-OE mice 2 weeks after MCAO (Figures S1 and S2). Taken together, these findings suggest that *Tlx* overexpression induced an increase in neurogenesis in SVZ, and the newly formed neuroblasts migrated toward the lesion site in experimental stroke.

### 2.3. Increased Number of Newborn Neurons in the Tlx-OE Mice Contributed to Better Motor Function Recovery After Stroke

**Figure 3:**
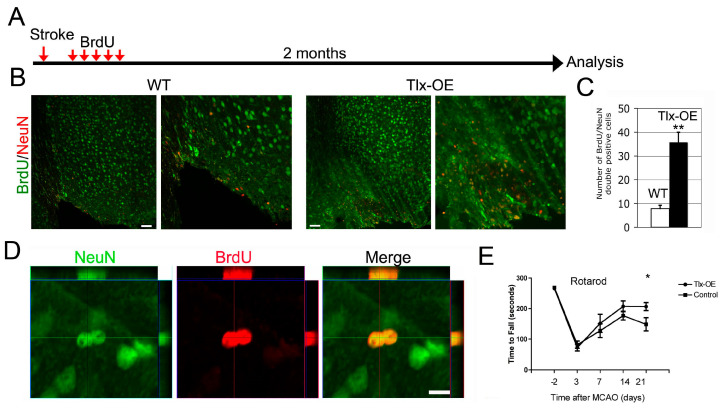
*Tlx* overexpression-mediated increase in stroke-induced neurogenesis results in increase in newly formed mature neurons. (**A**) Scheme of experimental procedure. (**B**) BrdU/NeuN costaining of mouse brain sections 2 months after stroke. Mice received continuous BrdU injection for 5 days 1 week after stroke. Scale bar: 50 µm. (**C**) Number of BrdU/NeuN double-positive cells per section in the cortex 2 months after stroke (*n* = 8, mean ± SD, ** *p* < 0.01). (**D**) Z-stack image of two adjacent BrdU/NeuN double-positive cells in the *Tlx*-OE cortex 2 months after stroke. Scale bar: 20 µm. (**E**) Rotarod test demonstrates that *Tlx*-OE mice recover better than control animals after stroke. (*n* = 14, * *p* < 0.05).

To investigate the number of newborn neurons of the *Tlx*-OE mice a long time after stroke, we performed the experiments described in Figure 3A. Only a few BrdU/NeuN-double-positive cells were observed in the damaged area of WT mice, and 4–5 times more BrdU/NeuN-positive cells were found in the *Tlx*-OE mice (Figure 3B,C). Z-stack images indicate that those cells that were BrdU/NeuN-positive appeared as pairs, suggesting that a division had happened before they differentiated into post-mitotic neurons (Figure 3D). These results directly demonstrate that the endogenous NSCs could be manipulated, and they contributed to neuronal repair significantly after stroke. To further investigate whether the increased neurogenesis had any effect on functional outcomes, we performed an experiment to measure the functional recovery of stroked animals. We observed that *Tlx*-OE mice recovered much better than control mice in the rotarod test (Figure 3E), which suggests that the newborn neurons in the *Tlx*-OE mice were contributing to the coordination and motor functional recovery after stroke.

### 2.4. Tlx Overexpression Led to an Increase in Stroke-Induced Neurogenesis in Aged Mice

**Figure 4:**
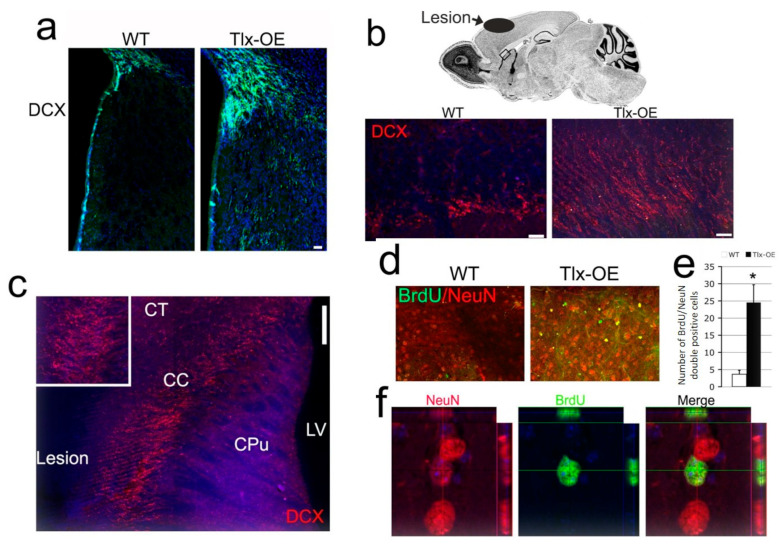
*Tlx* overexpression results in consistent stroke-induced neurogenic phenotype in aged mice. (**a**) DCX IHC of stroke brain sections (2 weeks) from 1 year old mice. Note that DCX-positive cells in the WT striatum are very rare. Scale bar: 50 µm. (**b**) DCX staining on sagittal sections of 1 year old mice 2 months after stroke; pictures acquired from the square region indicate the initial part of the rostral migratory stream. (**c**) Overview of DCX staining pattern described above. Note that the majority of DCX-positive cells are orientated toward the lesion area. (Inset showing a higher magnification). Scale bar: 100 µm. (**d**) BrdU/NeuN costaining of brain sections of approximately 1-year-old mice 3 months after stroke. Scale bar: 20 µm. (**e**) Number of BrdU/NeuN-double positive cells in striatum 3 months after stroke (*n* = 6, mean ± SD, * *p* < 0.05). (**f**) Z-stack image of a BrdU/NeuN double-positive cell in the *Tlx*-OE cortex 3 months after stroke. Scale bar: 20 µm.

To investigate the contribution of *Tlx*-overexpressing NSCs to stroke in aged animals, we performed pMCAO in 1-year-old mice. DCX staining was hardly detectable in the striatum of WT mice 2 weeks after pMCAO (Figure 4a), suggesting that there was a strong decrease in stroke-induced neurogenesis in aged mice. Many DCX-expressing cells were found in *Tlx*-OE mice (Figure 4a), which indicates that *Tlx* overexpression also strongly stimulated stroke-induced neurogenesis in aged animals. Surprisingly, 2 months after the stroke, the DCX staining of sagittal sections of the *Tlx*-OE mice with stroke demonstrated that the majority of DCX-positive cells were still migrating to the lesion through the corpus callosum (Figure 4b,c). The vast majority of the DCX-positive cells were orientated toward the damaged cortical area instead of to the OB (Figure 4c). We also analyzed the survival and maturation of the newly formed neurons in those mice 3 months after MCAO. Many BrdU/NeuN-positive cells were also found in the cortex (Figure 4d,f) and striatum (Figure 4e) of *Tlx*-OE mouse brains, whereas the number of newborn neurons was almost negligible in WT mice.

## 3. Discussion

Here, we report that the overexpression of *Tlx* gene increased neurogenesis after pMCAO. These newly formed neurons integrated into the existing network and contributed to the cellular recovery and improvement of the neurological score. After a stroke, the brain tissue is damaged, which in many post-stroke patients contributes to physical disabilities and impairment of motor and cognitive function. Although, as a consequence of ischemic stroke, new neurons are generated, most of them die and fail to integrate into the existing network [6,16]. Therefore, it is necessary to explore mechanisms that can promote neurogenesis and increase the survival of newly formed neurons. In this study, we investigated the contribution of *Tlx* gene overexpression to neurogenesis after experimental stroke in mice.

*Tlx* is expressed in neural stem cells in SVZ and SGZ [19]. The expression of *Tlx* was strongly upregulated in the SVZ of stroke-induced mice than in sham-operated mice (Figure 1a), suggesting the role of *Tlx* in increased SVZ neurogenesis after stroke. Previous studies have shown that *Tlx* contributes to adult neurogenesis through various mechanisms [19]. To confirm the contribution of *Tlx* to increased neurogenesis after stroke, we used *Tlx*^−/−^ mice. We observed a lack of neurogenesis in these *Tlx*^−/−^ mice after stroke (Figure 1b). This is consistent with previous findings in that the loss of *Tlx* led to a lack of adult neurogenesis [8,20]. Little evidence has demonstrated using genetic fate mapping the stem cell origin of newborn neurons after stroke. A previous longitudinal study showed newborn neurons from SVZ migrating toward and being present in the peri-infarct area [17]. However, our fate mapping results directly demonstrate that these newborn neurons and their progeny migrating toward the damaged tissue and differentiating into mature neurons in vivo were *Tlx*-positive cells (Figure 1c,d).

Although endogenous NSCs respond to brain injury by generating more neuroblasts toward the stroke site, the number of newborn cells is limited, and the majority of those cells die during their migration to the damaged area [6,16]. Thus, the stimulation of endogenous neurogenesis has been tested with many different approaches, and the most used is the infusion of growth factors or ligands, which lead to an increase in SVZ cell proliferation [15]; however, since most of the infused factors have other effects besides their role in neurogenesis, an NSC-specific stimulation approach is needed to evaluate the capacity of endogenous NSCs. In a previous study, a neural precursor-specific nestin promoter was used to drive the *Tlx* expression, which resulted in increased neural progenitor cell proliferation and enhanced hippocampal neurogenesis in mice [21]. We, however, used *Tlx*-overexpressing mice generated by bacterial artificial chromosome (BAC)-mediated transgenesis, which resulted in a two times higher expression of the *Tlx* gene under its own regulatory sequence. As we have shown before, the number of NSCs and the amount of neurogenesis in the SVZ of the *Tlx*-overexpressing (*Tlx*-OE) mice were increased, and this increase persisted even in 2-year-old mice [14]. We, therefore, asked whether the increase in NSCs contributes to cellular recovery in mice after stroke.

The experimental stroke in *Tlx*-OE mice showed that *Tlx* overexpression induced an increase in neurogenesis in SVZ (Figure 2d), and the newly formed neuroblasts migrated toward the lesion site, which was absent on the contralateral side and in sham-operated mice (Figure 2a–c,e). Previous studies have reported similar findings showing after stroke cells generated in SVZ adopt an altered path directed toward the lesion site [22]. The presence of increased neurogenesis only on the ipsilateral side of stroked animals suggests that this increase in neurogenesis is a stroke-associated phenomenon. Even after two months, more cells originated from SVZ neurogenic niche were observed in *Tlx*-OE mice than in WT mice (Figure 3B,C), suggesting a constitutive contribution of neurogenesis to neuronal repair after stroke. Subsequently, we performed behavioral analysis to investigate if this observed cellular recovery translates into the improvement of functional performance. The *Tlx*-OE mice showed better recovery than control mice in the rotarod test (Figure 3E), which suggests that the newborn neurons in the *Tlx*-OE mice were contributing to the coordination and motor functional recovery after stroke, agreeing with the findings of previous studies that *Tlx* overexpression improves behavioral score and cognitive deficits in rats and mice [11,21,23].

Inflammation and oxidative stress contribute heavily to outcomes following ischemic stroke [24,25,26]. Clinical studies have associated oxidative stress and pro-inflammatory cytokines such as IL-1β, IL-6, and TNF-α with poor outcomes in ischemic stroke patients [27,28,29]. Experimental animal studies have suggested that blocking NF-κB activation and suppressing the expression of these pro-inflammatory cytokines can reduce brain tissue damage and improve behavioral scores and cognitive deficits [30,31]. Interestingly, *Tlx* has been shown to suppress the pro-inflammatory NF-κB pathway and ameliorate the expression of IL-1β and TNF-α [11], which could be an alternative mechanism, in addition to improved neurogenesis, underlying a better behavioral score. Furthermore, *Tlx*, by suppressing oxidative stress [10] and improving the expression of SIRT1 [11], can also contribute to better outcomes after experimental ischemia [26,32]. Taken together, it can be suggested that *Tlx* overexpression can contribute to increased neurogenesis, promote cellular recovery, and improve functional recovery after stroke.

One of the limitations of most mouse stroke models is that the experiments are performed in young adult animals, whereas stroke mostly affects aged people, and it has been shown that the risk of stroke doubles every decade from the age of 55 [33]. It is known that the number of NSCs in the SVZ declines with age [34,35], and it has been shown that stroke-induced neurogenesis (1 h MCAO) is reduced in aged rats [36], although another study disagrees with these results [37]. In this context, it is important to recall that *Tlx* overexpression led to a long-term increase in neurogenesis (Figure 3). In one-year-old mice, an increased number of newborn neurons were observed in the *Tlx*-OE than WT mice (Figure 4). In *Tlx*-OE mice, newborn neurons migrating toward the damaged area could be observed after two months and three months (Figure 4). These findings advocate that targeting *Tlx* can be beneficial after stroke even for old patients.

## 4. Materials and Methods

### 4.1. Animals

Mice were housed according to standard conditions, and all animal experiments conformed to local and international guidelines for the use of experimental animals. The *Tlx* conventional knock-out mice, *Tlx*-CreER^T2^ mice, and *Tlx*-OE mice used were described before [8,14,38]. Briefly, BAC technology was utilized to achieve an accurate expression of the tamoxifen-inducible Cre recombinase (CreER^T2^ fusion protein) controlled by the *Tlx* gene regulatory sequences. Through homologous recombination in Escherichia coli, a section of exon 1 of the *Tlx* gene was substituted with the CreER^T2^ cassette. The modified BAC will produce CreER^T2^ under the regulation of *Tlx* gene regulatory sequences found within 104 kb of 5′ flanking sequences and 22 kb of 3′ flanking sequences. The tamoxifen-induced Cre recombinase translocation was tested in the F1 offspring of six founders. Cre recombinase consistently resided in the cytoplasm before treatment and moved to the nucleus after tamoxifen injection. Consequently, two copies of the transgenes were expressed. After tamoxifen injection, efficient Cre translocation to the nucleus was observed, and the Cre expression pattern was similar to that of the natural *Tlx* gene. Z/EG mice were obtained from the Jackson Lab. Experiments were approved by the North Rhine–Westphalia State Agency for Nature, Environment and Consumer Protection (approval number 84-02.04.2012.A120).

### 4.2. MCAO Models

#### 4.2.1. Permanent Middle Cerebral Artery Occlusion

Male mice were anesthetized by performing an i.p. injection of 150 µL 2.5% tribromoethanol per 10 g body weight. A skin incision was made between the ear and the orbit on the left side. The parotid gland was removed by electrical coagulation. The stem of the MCA was exposed through a burr hole and occluded by microbipolar coagulation (Erbe, Tübingen, Germany). Surgery was performed under a microscope (Hund, Wetzlar, Germany). A body temperature of 37 °C was maintained in the mice using a heating pad. After the time indicated, mice were deeply reanesthetized with tribromoethanol and perfused intracardially with Ringer’s solution.

#### 4.2.2. Transient Middle Cerebral Artery Occlusion

Mice were anesthetized with isoflurane (2%) and N_2_O/O_2_ (70%/30%). For middle cerebral artery occlusion (MCAO), a medial neck incision was performed, and the left common carotid artery was exposed. A monofiler Nylonfilament of size 7-0 (Fa. Doccol, Katalog-Nr.7019PK5Re) was inserted into the common carotid artery on the left side. It was advanced into the internal carotid artery around 11 mm from the bifurcation, and the successful occlusion was monitored by measuring considerable drops in the laser Doppler signal. The filament was reversibly fixed during the whole period of ischemia. Surgery was performed under a microscope (Hund, 10 Wetzlar, Germany), and the body temperature of 37 °C was maintained using a heating pad. Mice were reanesthetized, and the filament was removed after 45 min of ischemia. For laser Doppler measurements, the probe (P415-205; Perimed, Piscataway, NJ, USA) was placed 3 mm lateral and 6 mm posterior to the bregma. Relative perfusion units were determined (Periflux 4001; Perimed AB, Järfälla, Sweden).

The transient middle cerebral artery occlusion (tMCAO) model is well established for long-term behavioral analysis [39]. Because of the high mortality in the tMCAO model, we reduced the ischemia duration to 35 min of ischemia and reperfusion, which mainly leads to a striatal lesion. Behavioral tests were performed on day 2, d3, d7, d14, and d21.

### 4.3. Behavioral Analysis

All behavioral tests were performed blindly, and mice were randomized. Mice were kept under standard condition in separate cages and habituated for 4 days.

### 4.4. Rotarod

The rotarod test was used to assess the motor deficits. Mice were trained on a mouse rotarod (kat. No. 47600, ugo basile, Comero, Italy) for 3 sessions with a speed of 16 rpm before MCAO. For the experiment, we used a speed with increasing velocity from 0 rpm to a maximum of 40 rpm. Each experiment was repeated 3 times, and the mean time to stay on the rotarod was measured.

### 4.5. BrdU Administration and Tamoxifen Injection

BrdU (Sigma, St. Louis, MO, USA) was dissolved in sterile NaCl to prepare 15 mg mL^−1^ solution, and mice were injected intraperitoneally with 50 mg kg^−1^ as indicated, respectively, in the figures. Tamoxifen (Sigma) was dissolved in sunflower seed oil (Sigma) with 10% EtOH_abs_ to prepare the 10 mg mL^−1^ solution. Tamoxifen was injected for 5 days, 2 times/day in *Tlx*-CreER^T2^;Z/EG mice or the littermate control mice.

### 4.6. Immunohistochemistry

Mice were perfused with 4% paraformaldehyde, and the brains were postfixed overnight at 4 °C. 50 µm vibratome sections or 5 µm paraffin sections were blocked in 5% normal swine serum in PBST (PBS + 0.2%, Triton-X 100) and incubated overnight at 4 °C with the primary antibody. The dilutions of primary antibodies used in this study were as follows: *Tlx* (rabbit, 1:500, Homemade); GFP (rabbit, 1:1000, Molecular Probes, Thermo Fisher Scientific, Waltham, MA, USA); Doublecortin (goat, 1:100, Santa Cruz, Heidelberg, Germany); BrdU (mouse, 1:50, Dako, Hamburg, Germany; sheep, 1:100, Abcam, MA, USA); NeuN (mouse, 1:400, Chemicon, IL, USA). In controls in which the primary antibody was omitted, no immunostaining was seen. The visualization of primary antibodies was performed with either a horseradish peroxidase system (ABC kit, Vector, CA, USA) or immunofluorescence with Alexa 488 or Alexa 594 (1:100, Molecular Probes, Thermo Fisher Scientific, OR, USA). The secondary antibodies for costainings were conjugated with Alexa 350, Alexa 488, and Alexa 594 (1:100, Molecular Probes). Fluorescent images were captured in 1.5 mm optical sections using a confocal laser-scanning microscope (LSM710, Zeiss, Oberkochen, Germany).

### 4.7. Statistical Analysis

The data were analyzed in prism. For two groups, *t*-test was used and for more than two groups, One-Way ANOVA followed by TUKEY’s test was used. The significance level was set at *p* < 0.05.

## 5. Conclusions

In conclusion, these results provide direct in vivo evidence that NSCs significantly contribute to the neuronal regeneration of stroke-induced cell loss in young and aged animals, thus strongly suggesting that the strategy is applicable to induce endogenous neurogenesis after stroke. These observations in the context of our previous findings indicate that attempts to increase *Tlx* activity might be very attractive as a specific remediant for stroke-induced neural deficiencies. As a nuclear receptor, *Tlx* is a druggable protein and can serve as a very effective therapeutic target once its ligand is identified.

## 6. Limitations

In this study, we could not investigate the effects of *Tlx* overexpression on cellular degeneration and apoptosis after experimental stroke. Also, our study does not include the behavioral analysis for cognitive and memory functions.

## Data Availability

The original contributions presented in the study are included in the article/Supplementary Material; further inquiries can be directed to the corresponding author.

## Supplementary Materials

The following supporting information can be downloaded at: https://www.mdpi.com/article/10.3390/ijms252212440/s1.

## Abbreviations

SVZ	subventricular zone (SVZ)
NSCs	neural stem cells
LV	lateral ventricle
SGZ	subgranular zone
DG	dentate gyrus
MCAO	middle cerebral artery occlusion
tMCAO	transient middle cerebral artery occlusion
pMCAO	permanent cortical middle cerebral artery occlusion
WT	wild type
DCX	doublecortin
