# Supplementary material for: Tlx Promotes Stroke-Induced Neurogenesis and Neuronal Repair in Young and Aged Mice
Source: Int J Mol Sci. 2024 Nov 19;25(22):12440. doi: 10.3390/ijms252212440 (PMC11594625; doi:10.3390/ijms252212440)
Supplement: Supplementary file 1 [file ijms-25-12440-s001.zip › ijms-3285001-supplementary.pdf]

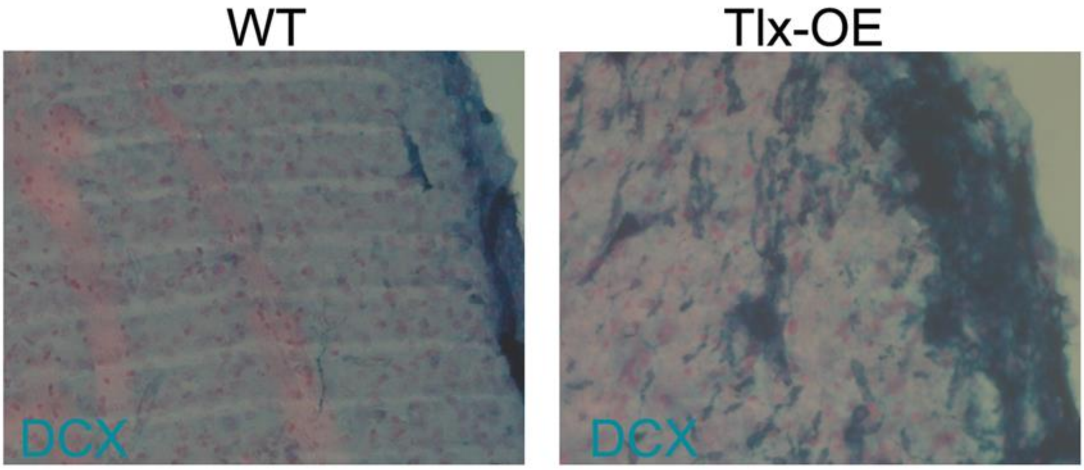

Figure S1. DCX antibody staining of brain sections from *Tlx*-OE mice 2 weeks after stroke (half hour transient MCAO). Note that only few DCX-positive cells are found in WT brain sections.

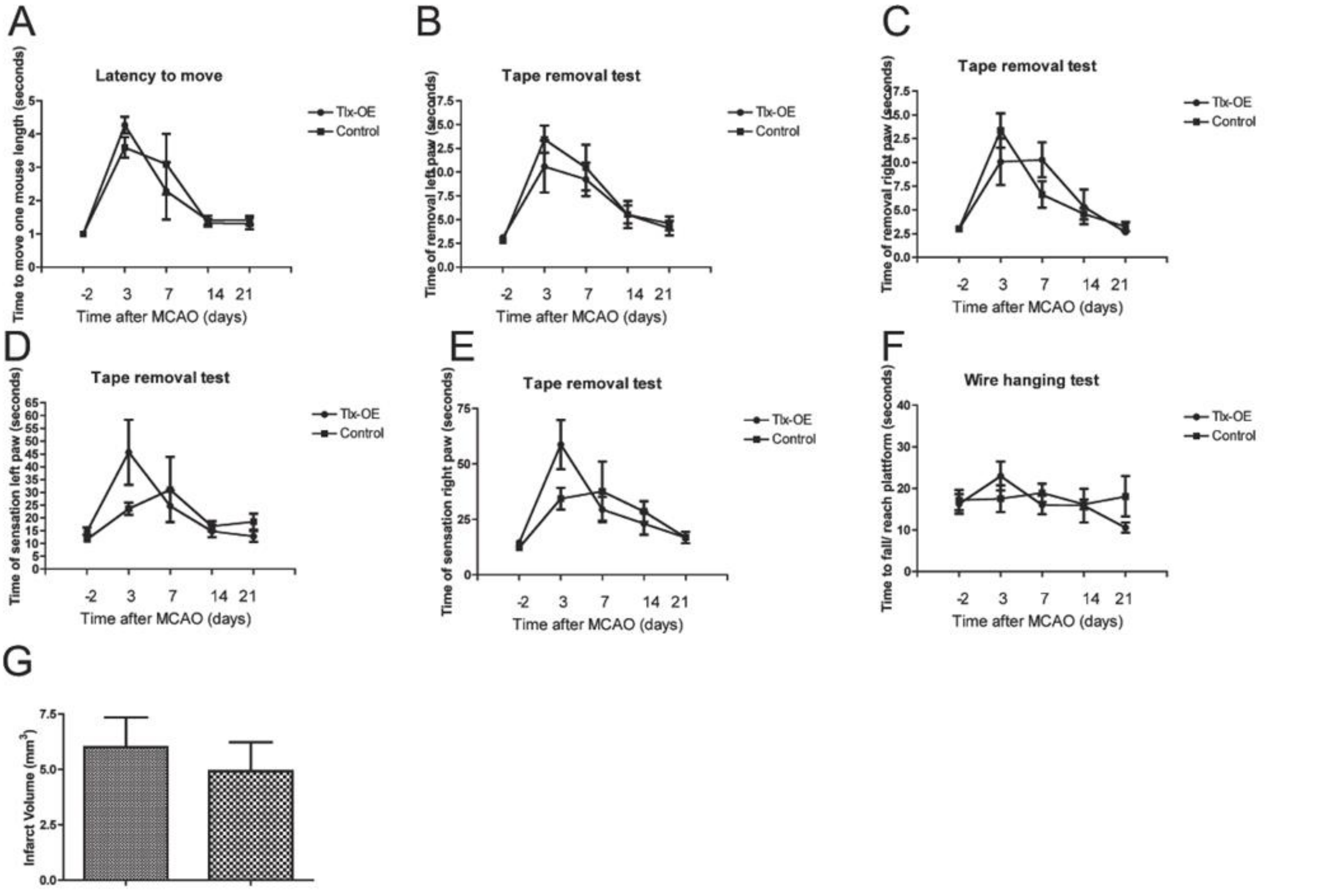

Figure S2. No significant differences were observed between Tlx-OE and control mice using a panel of behavioral tests: (A) Latency to move, (B-E) Tape removal and sensation test, (F) Wire hanging test. (G) No difference of infarct volume was observed between Tlx-OE and control mice.
